# Noninvasive prenatal testing for fetal subchromosomal copy number variations and chromosomal aneuploidy by low‐pass whole‐genome sequencing

**DOI:** 10.1002/mgg3.674

**Published:** 2019-04-19

**Authors:** Dongyi Yu, Kai Zhang, Meiyan Han, Wei Pan, Ying Chen, Yunfeng Wang, Hongyan Jiao, Ling Duan, Qiying Zhu, Xiaojie Song, Yan Hong, Chen Chen, Juan Wang, Feng Hui, Linzhou Huang, Chongjian Chen, Yang Du

**Affiliations:** ^1^ Genetic Testing Center, Qingdao Women & Children Hospital Qingdao University Qingdao China; ^2^ The Center of Prenatal Diagnosis Affiliated Hospital of Guizhou Medical University Guiyang China; ^3^ Central Lab Wuxi Maternity and Child Health Care Hospital, Affiliated Wuxi Maternity and Child Health Care Hospital of Nanjing Medical University Wuxi, Jiangsu China; ^4^ Annoroad Gene Technology Co., Ltd Beijing China; ^5^ The Branch Center of Prenatal Diagnosis Hebei Maternity and Child Healthcare Hospital Shijiazhuang China; ^6^ Department of Obstetrics First Affiliated Hospital of Xinjiang Medical University Urumqi China; ^7^ Department of Obstetrics and Gynecology Wuhan Women and Child Care Service Hospital Wuhan China

**Keywords:** cell‐free DNA, chromosome aneuploidy, NIPSCCD, NIPT, subchromosomal CNVs

## Abstract

**Background:**

Expanding noninvasive prenatal testing (NIPT) to include the detection of fetal subchromosomal copy number variations (CNVs) significantly decreased the sensitivity and specificity. Developing analytic pipeline to achieve high performance in the noninvasive detection of CNVs will largely contribute to the application of CNVs screening in clinical practice.

**Methods:**

We developed the Noninvasively Prenatal Subchromosomal Copy number variation Detection (NIPSCCD) method based on low‐pass whole‐genome sequencing, and evaluated its efficacy in detecting fetal CNVs and chromosomal aneuploidies with 20,003 pregnant women.

**Results:**

Totally, NIPSCCD identified 36 CNVs, including 29 CNVs consistent and 7 CNVs inconsistent with amniocytes tests. Additionally, seven fetal CNVs identified by amniocytes testing were undetected by NIPSCCD. The sensitivities for detecting CNVs > 10 Mb, 5 Mb–10 Mb, and CNVs < 5 Mb were 91.67%, 100.00%, and 68.42%, respectively. Moreover, NIPSCCD identified 103/ true positive trisomy 21/18/13 cases and 21 false positives, producing an overall 100.00% sensitivity and 99.89% specificity.

**Conclusion:**

NIPSCCD showed a good performance in detecting fetal subchromosomal CNVs, especially for CNVs >10 Mb, and can be incorporated into the routine NIPT chromosomal aneuploidies screening with high sensitivity and specificity.

## 1 INTRODUCTION

The discovery of cell‐free fetal DNA (cffDNA) in maternal circulation and the rapid development of next‐generation sequencing enable the noninvasive detection of fetal genetic disorders using maternal plasma (Chiu et al., 2008; Fan, Blumenfeld, Chitkara, Hudgins, & Quake, 2008; Lo et al., 1997). In recent years, noninvasive prenatal testing (NIPT) has been successfully used for the screening of common fetal aneuploidies including trisomy 21 (Down's syndrome), trisomy 18 (Edward's syndrome), and trisomy 13 (Patau syndrome) with high sensitivity and specificity (Benn, Cuckle, & Pergament, 2013; Minear, Lewis, Pradhan, & Chandrasekharan, 2015; Taylor‐Phillips et al., 2016). However, it remains difficult to detect chromosome structural variations with conventional NIPT method. Subchromosomal copy number variations (CNVs), known as segmental deletions and duplications, are extensively distributed in human genome (Zarrei, MacDonald, Merico, & Scherer, 2015). A substantial proportion of these CNVs are associated with severe diseases such as Prader‐Willi/Angelman syndromes, Cri‐du‐chat syndrome (5p‐), DiGeorge syndrome (22q11), 1p36 deletion syndrome (Weise et al., 2012), and some newly identified microdeletion and microduplication syndromes (Goldenberg, 2018; Nevado et al., 2014). It has been reported that clinically relevant CNVs occur in 6% of fetuses with a structural or growth anomalies (Wapner et al., 2012). Distinguished from chromosomal aneuploidies, the incidence of CNVs is independent of maternal age (Wapner et al., 2015). Thus, the prenatal detection of CNVs with clinical significance would benefit all the pregnant women in genetic counseling and clinical management of the pregnant outcomes. As a result, the expansion of NIPT from detection of chromosomal aneuploidies to subchromosomal CNVs has long attracted the attention of clinical investigators.

Previous studies have explored the feasibility of whole‐genome sequencing (WGS) based NIPT in fetal CNVs detection (Benn & Cuckle, 2014; Peters et al., 2011; Srinivasan, Bianchi, Huang, Sehnert, & Rava, 2013; Yu et al., 2013), but the requirement of deep sequencing made it far from cost‐efficient for clinical practice. Therefore, bioinformatic pipelines based on low‐coverage sequencing of whole genome with maternal plasma were developed to solve this problem. Chen et al. demonstrated that the Fetal Copy‐number Analysis through Maternal Plasma Sequencing (FCAPS) method could be used to detect large subchromosomal deletions and duplications in fetuses at a 0.08 × sequencing depth (Chen et al., 2013). Further study using a collection of 919 clinical samples with known CNVs suggested that the FCAPS method is of high sensitivity and specificity in detecting CNVs larger than 10 Mb (Liu et al., 2016). Straver et al. proposed a WIthinSamplE COpy Number aberration DetectOR (WISECONDOR) method that could detect CNVs down to 20 Mb in size at the sequencing depth of 0.15–1.66× (Straver et al., 2014). Moreover, by using the semiconductor sequencing platform (SSP), 71.8% of the fetal CNVs ranging from 0.52 Mb to 84 Mb in size could be detected with 3.5 M reads, but the sensitivity declined to 41.2% when the CNVs sizes were restricted between 1 Mb and 5 Mb (Yin et al., 2015). Focusing on the removal of sequencing “noise”, Zhao et al. developed a novel method using shallow WGS (approximately 0.2×) which correctly identified 17 of 18 cases with microdeletions/microduplications and 156 of 157 unaffected cases (Zhao et al., 2015). It was reported that 15 out of 18 samples with CNVs larger than 6 Mb could be detected at read counts between 4 M and 10 M using a calling pipeline based on segmentation algorithm. However, only 4 out of 13 CNVs were correctly identified if the CNV was smaller than 6 Mb (Lo et al., 2016).

To date, it remains challenging to detect small CNVs by NIPT because the proportion of cffDNA involved in the imbalanced chromosomal segments is limited (Rose, Benn, & Milunsky, 2016). Despite of the fact that commercial providers have offered NIPT analysis for certain pathogenic CNVs, the use of NIPT in detecting microdeletions and microduplications has not reached an acceptable false positive rate to be deemed practical in clinical setting (Advani, Barrett, Evans, & Choolani, 2017). In this study, we developed the Noninvasively Prenatal Subchromosomal Copy number variation Detection (NIPSCCD) method, for the genome‐wide analysis of fetal CNVs through low‐pass WGS of maternal plasma, and evaluated its performance in 20,003 successfully tested clinical samples. Our study demonstrated that the NIPSCCD method could be incorporated into the current NIPT program for fetal chromosomal aneuploidy screening and it has good performance in the detection of fetal CNVs.

## 2 MATERIALS AND METHODS

### 2.1 Editorial policies and ethical considerations

This study was approved by the Ethics Committee of hospitals including Qingdao Women & Children Hospital of Qingdao University, Affiliated Hospital of Guizhou Medical University, Wuxi Maternal and Child Health Care Hospital, Hebei Maternity and Child Healthcare Hospital, First Affiliated Hospital of Xinjiang Medical University and Wuhan Women and Child Care Service Hospital. Informed consents were obtained from all individual participants included in the study.

### 2.2 Sample collection

A total of 20,290 pregnant women were recruited with informed consents between 30 July 2015 and 30 June 2016 from several provinces in China, including Shandong, Hebei, Hubei, Jiangsu, and Sinkiang. Ethical approvals were granted by the Ethics Committee of hospitals enrolled in this study. NIPSCCD was offered to the participants as primary or secondary screening. Since the objective of the study was to report the performance of NIPSCCD in detecting subchromosomal CNVs as well as trisomy 21, 18, and 13, other chromosomal abnormalities including monosomies and other trisomies were screened but not analyzed. Each participant received counselling after NIPSCCD screening. Positive NIPSCCD individuals with trisomy 21, 18, and 13 were suggested to undergo invasive testing for prenatal diagnosis, while positive individuals with CNVs detected by NIPSCCD were validated by amniocentesis followed by whole‐genome sequencing (Qi et al., 2018) or telephone follow‐ups. All the negative ones were confirmed by telephone follow‐ups. Invasive testing and follow‐up results were used as gold standard to calculate the sensitivity and specificity of NIPSCCD. For all NIPSCCD tests, 10‐ml peripheral blood from individual was collected in EDTA‐containing tubes (Sekisui, Tokyo, Japan). The plasma was separated within 4 hr after blood sample collection with previously described method (Yin et al., 2018) and stored at −80°C for further analysis.

### 2.3 Library construction and DNA sequencing

The genomic DNA isolated from amniocytes was fragmented into an average size of 250 bp, whereas cell‐free DNA (cfDNA) was isolated with MagMAX™ Cell‐Free DNA Isolation Kit (Applied Biosystems™ cat.: A29319) according to the manufacture's instruction. Library construction, quality control, and sequencing were performed as previously described (Liang et al., 2013; Qi et al., 2018; Song et al., 2013). Briefly, 2.5 ng of cfDNA or fragmented DNA was used for the preparation of sequencing libraries. The 8‐bp barcoded sequencing adaptors were ligated to fragments and amplified by PCR. Purified libraries were sequenced using NextSeq 550AR (Annoroad Gene Technology Co., Ltd China). For the confirmatory tests, an average of 7.5‐M reads with 40 bp in length was generated for each amniocytes sample, accounting for 0.1× of the human genome; for each maternal plasma sample, an average of 4.2‐M reads with 40 bp in length and Q30 > 95% was generated for further analysis. Samples that failed to pass the in‐house quality control of cfDNA testing in the first round were subjected to repeat testing. In total, 20,232 samples had successful NIPSCCD results after the second test. For the evaluation of the performance of NIPSCCD, 20,003 successfully tested samples with confirmatory results or follow‐up results were used.

### 2.4 Data analysis

All short sequencing reads were aligned to human reference genome (GRCh37/hg19) with BWA aligner (Li & Durbin, 2009). Unique reads were counted in each of the nonoverlapping 100‐Kb windows that were equally divided along the chromosomes. Reads count within each 100‐Kb window was corrected with a LOWESS model using GC content per window, and capped at the mean plus or minus 3 times of the residual standard deviation when the corrected value exceeded the two capping thresholds. The corrected values were then transformed into raw *Z*‐scores with mean 0 and standard deviation of 1.

$$Z_{\text{raw},\text{i}}=\text{lowess}\left({\text{Reads}}_{i},{\text{GC}}_{i}\right)$$

$$Z_{\text{wg},i}=\frac{Z_{\text{raw},i}-\text{Mean}\left(Z_{\text{raw}}\right)}{\text{Sd}\left(Z_{\text{raw}}\right)}$$

A set of 1,000 reference samples were preselected from a separate group of low risk NIPT samples without chromosomal aneuploidies and pathogenic CNVs. Read counts for this set of data were similarly processed using the correction model.

For each testing sample, an additional round of *Z*‐score transformation was performed for each 100‐Kb window by subtracting the mean value of the raw *Z*‐scores of the corresponding windows from the reference set, and then divided by their standard deviation.

$$Z_{i}=\frac{Z_{\text{wg},i}-\text{Mean}\left(Z_{\text{ref}}\right)}{\text{Sd}\left(Z_{\text{ref}}\right)}$$

To obtain the confidence interval for the estimated *Z*‐scores, these transformations were also performed for the reference samples using bootstrap resampling. Windows were merged into continuous segments by applying the CBS algorithm to obtain the final *Z*‐scores (Venkatraman & Olshen, 2007). Subchromosomal segments with absolute values of the final *Z*‐scores above 1.28 were considered indicative of CNVs, while z‐scores of a whole chromosome above 3 were considered as trisomy 21/18/13. For the borderline *Z*‐score samples, a retest was carried out to confirm the results. The significance levels of the detected segments were evaluated using the distribution of those final *Z*‐scores of the covered windows in the reference set.

**Figure 1:**
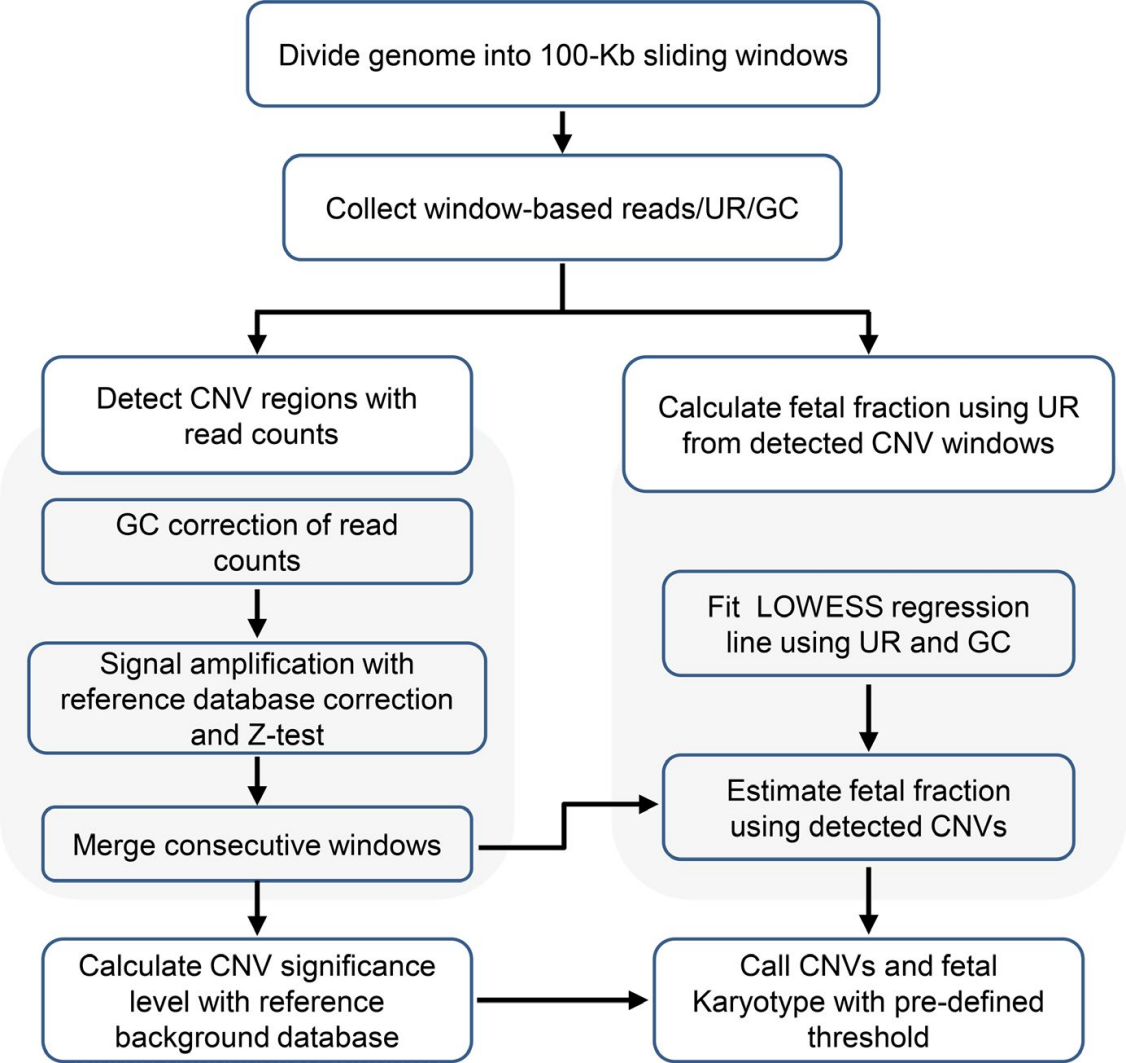
The workflow of the NIPSCCD method. UR, unique reads

Fetal fraction estimation based on reads mapped to chromosome Y was conducted as previously reported (Zhang, Zhao, et al., 2015). Within the detected CNVs region, an alternate estimation of fetal fraction was also performed using a depth‐based model similar to the way of chromosome Y based fetal fraction estimation. CNV events were filtered out as false positives when the deviation between two fetal fraction estimations was larger than 3 times of the standard deviation of the reference set (internally constructed using validated aneuploidy male fetus). The workflow of the NIPSCCD was summarized as Figure 1.

## 3 RESULTS

### 3.1 Study population

To evaluate the performance of NIPSCCD method (Figure 1) for fetal subchromosomal CNVs and trisomy 21/18/13 screening, we conducted the low‐pass WGS (about 4.2 M reads) using peripheral blood samples of 20,290 pregnant women. Cell‐free DNA (cfDNA) testing was successfully performed on 19,805 (97.61%) of 20,290 cases in the first round and 485 (2.39%) samples failed the in‐house quality control, in which 95 (0.47%) cases required repeat blood sampling. After the second testing, 427 of 485 women were successfully tested and 58 women who gave noninformative results were excluded. Therefore, a total of 20,232 (99.71%, 20,232/20,290) women had successful NIPSCCD results.

Out of the 20,232 pregnant women, 229 cases were excluded from further analysis, including 152 cases with NIPSCCD positive results of monosomies and trisomies beyond trisomy 21/18/13, 27 cases with CNVs of unknown clinical significance but refused to undergo amniocentesis, and 50 cases due to the loss of contact. Finally, 20,003 samples with confirmatory results or follow‐up data were used for the evaluation of NIPSCCD performance. The mean maternal age was 32 years ranging from 28 to 48 years, and the mean gestational week was 18.2, ranging from 11 to 20 weeks (Table 1).

**Table 1:** Demographic characteristics of the samples in this study

Body index	Range (Min–Max)	Mean ± *SD*
Maternal age	28.0–48.0	32.2 ± 5.3
Gestational weeks	11.0–20.0	18.2 ± 2.8
Maternal weight (kg)	45.0–79.8	60.6 ± 10.3

### 3.2 Evaluation of NIPSCCD for fetal CNVs detection

In 20,232 pregnancies with successful cfDNA testing, the NIPSCCD method identified 64 samples with single or multiple CNVs and produced a total number of 72 CNVs. Due to the refusal of invasive procedures, additional confirmatory results were obtained for 36 NIPSCCD‐identified CNVs across 28 pregnant women. Moreover, seven additional CNVs were not detected by the NIPSCCD but by amniocentesis testing. The detailed information of the CNVs were listed in Tables 2 and 3.

**Table 2:** Consistent CNVs detected by the NIPSCCD method and amniocytes testing in this study

Sample ID	Fetal gender	GW	Amniocytes testing (AT)		NIPSCCD results	Location accuracy	FF by ChrY	Pathogenicity^a^
			Location	Size (Mb)				
EC13CD00073	Female	20 + 5	Dup 4q28.1‐4q35.2	66.10	Dup 4q27‐4q35.2	Covered the AT	NA	NA
			Del 5p15.33‐5p14.1	26.55	Del 5p15.33‐5p14.1	99% covered by AT (26.3 Mb)		Cri‐du‐chat syndrome
EC13SA00154	Female	24 + 6	Dup 12p13.33‐12q11	37.80	Dup 12p13.33‐12q12	Covered the AT	NA	NA
			Dup 10q25.1‐10q25.1	1.05	Dup 10q25.1‐10q25.2	Covered the AT		NA
EC13EX00442	Female	19	Del 5p15.33‐5p14.3	20.70	Del 5p15.33‐5p14.3	Covered the AT	NA	Cri‐du‐chat syndrome
			Dup 9p24.3‐9p23	11.20	Dup 9p24.3‐9p23	Covered the AT		NA
			Dup 9p23‐9p22.3	1.00	Dup 9p23‐9p22.3	Covered 70% of the AT (0.7 Mb)		NA
EC13BD00154	Female	22 + 3	Dup 18q21.32‐18q23	20.60	Dup 18q21.32‐18q22.3	97% covered by AT (14.55 Mb)	NA	NA
			Del 18p11.32‐18p11.31	4.75	Del 18p11.32‐18p11.21	Covered the AT		Chromosome 18p deletion syndrome
EC13BK00001	Female	21 + 5	Del 5q35.2‐5q35.3	7.05	Del 5q35.3‐Del 5q35.3	97% covered by AT (3.0 Mb)	2.55%	NA
			Dup 7q36.1‐7q36.3	6.55	Dup 7q36.1‐7q36.3	Covered the AT		NA
EC13BK00003	Female	20 + 5	Dup 16q24.1‐16q24.3	5.35	Dup 16q24.1‐16q24.3	Covered by AT	NA	NA
			Del Xp22.33‐Xp22.31	3.80	Del Xp22.33‐Xp22.31	83% covered by AT (3.6 Mb)		NA
EB13EX00305	Female	20 + 2	Dup 2p21‐2p21	0.80	Dup 2p21‐2p16.3	Covered the AT	NA	NA
			Dup Xp22.13‐Xp22.12	0.80	Dup Xp22.13‐Xp22.12	Covered the AT		NA
EC13CD00028	Female	19 + 3	Del Xp22.33‐Xp11.1	58.05	Del Xp22.33‐Xq11.2	Covered the AT	NA	Multiple diseases^b^
EC13EX01294	Male	13 + 5	Dup 11q14.3‐11q25	43.25	Dup 11q14.3‐11q25	98% Covered by AT (41.20 Mb)	15.91%	NA
EC13BK00006	Male	21 + 3	Dup 12p13.33‐12p11.1	34.75	Dup 12p13.33‐12p11.21	Covered by AT	5.19%	NA
EC17QD00108	Male	17 + 2	arr 5p15.33p13.2 (113576–34046734)×1	33.93	Del 5p15.33‐5p13.3	Covered by the AT	8.93%	Cri‐du‐chat syndrome
EC176L00184	Female	13 + 2	arr 18q22.1q23 (63578361–78013728)×1	14.44	Del 18q22.1‐18q23	Covered by the AT	NA	NA
EC13HB00814	Female	12 + 1	arr 5p15.33p15.2 (3222579–10164617)×1	6.95	Del 5p15.33‐5p15.2	Covered by the AT	NA	Cri‐du‐chat syndrome
EC16BE00322	Male	17 + 3	arr 15q11.2q13.1 (23290787–28526905)×3	5.24	Dup 15q11.2‐15q13.1	96% Covered by AT (4.90 Mb)	5.12%	15q11‐q13 duplication syndromes
EC13HB01069	Male	18	Dup 22q11.21‐22q11.21	2.60	Dup 22q11.21‐22q11.22	73% covered by AT (2.35 Mb)	8.05%	22q11duplication syndrome
EC13JD00020	Male	21	Dup 3q26.1‐3q26.1	2.55	Dup 3q26.1‐3q26.1	Covered the AT	16.02%	NA
EC13HB01084	Male	16 + 3	Dup 22q11.21‐22q11.21	2.48	Dup 22q11.21‐22q11.21	Covered the AT	14.38%	22q11 duplication syndrome
EC13AN00018	Female	26 + 3	Del 22q11.21‐22q11.23	2.35	Del 22q11.21‐22q11.23	Covered the AT	NA	22q11.2 distal deletion syndrome
EC13HB01354	Male	15 + 2	Del 1q21.1‐1q21.2	1.80	Del 1q21.1‐1q21.2	51% covered by AT (0.90 Mb)	12.34%	1q21.1 microdeletion syndrome
EC17HB42450	Male	24 + 6	arr 22q13.31q13.32 (47978041–49100597)×1	1.12	Del 22q13.31‐22q13.33	Covered the AT	9.88%	NA
EC178L04039	Male	20	Del 15q11.2‐15q11.2	0.36	Del 15q11.1‐15q11.2	Covered the AT	8.89%	Prader‐Willi/Angelman syndrome

Note

GW, gestation weeks; FF, fetal fraction estimated with reads mapped chromosome Y.

a Data from OMIM database (https://omim.org/) or DECIPHER database (https://decipher.sanger.ac.uk/); NA, not applicable.

b Multiple diseases including Leri‐Weill dyschondrostosis (LWD) ‐ SHOX deletion, Steroid sulphatase deficiency (STS).

**Table 3:** Inconsistent CNVs between the NIPSCCD and amniocytes testing results

Sample ID	Fetal gender	GW	Amniocytes testing (AT)		NIPSCCD results		FF by ChrY
			Location	Size (Mb)	Location	Size (Mb)	
EC13CD00028	Female	19 + 3	Del 12q23.1‐12q23.1	0.35	Undetected	NA	NA
			Dup Xp11.21–Xq28	97.20	Undetected	NA	
EC13EX00442	Female	19	Del 5p14.1‐5p14.1	2.35	Undetected	NA	NA
EC13AK00635	Female	19 + 2	Dup Xp22.31‐Xp22.31	1.75	Undetected	NA	NA
EC13CD00073	Female	20 + 5	Dup 7p21.3‐7p21.3	0.50	Undetected	NA	NA
EC13BD00154	Female	22 + 3	Del 2p14‐2p14	0.25	Undetected	NA	NA
EC178L04039	Male	20	Dup 22q11.22‐22q11.22	0.20	Undetected	NA	8.89%
EC17EU00255	Female	17 + 5	46, XX	NA	Del 18p11.32‐18p11.21	15.05	NA
EC13BK00007	Male	18 + 5	46, XY	NA	Dup 8q22.1‐8q23.1	11.10	5.54%
EC13EL00772	Male	14 + 4	46, XY	NA	Del 15q11.2‐15q13.1	7.25	8.57%
EC13HB01264	Male	16 + 1	46, XY	NA	Dup 8p23.1‐8p22	6.15	7.89%
EC13CD00105	Male	20 + 4	46, XY	NA	Del 17q25.3‐17q25.3	4.75	3.77%
EC13CD00086	Female	21 + 2	46, XX	NA	Dup 16p13.3‐16p13.2	0.85	NA
EC13JD00020	Male	21	NA	NA	Dup 4q32.2‐4q32.3	0.75	16.02%

Note

GW, gestation weeks; FF, fetal fraction estimated with reads mapped chromosome Y; NA, not applicable.

In total, the amniocytes testing identified 36 CNVs with the size ranging from 0.25 Mb to 97.2 Mb. There were 12 CNVs larger than 10 Mb, 5 CNVs between 5 Mb to 10 Mb, and 19 CNVs less than 5 Mb. Among these, 12 CNVs were found to be pathogenic including four Cri‐du‐chat syndrome related CNVs because of the 5p15.33‐5p14.1 deletion in sample EC13CD00073, 5p15.33‐5p14.3 deletion in sample EC13EX00442, 5p15.33‐p13.2 deletion in sample EC17QD00108, and the 5p15.33‐p15.2 deletion in sample EC13HB00814; two cases of 22q11 duplication syndrome caused by the duplication of 22q11.21‐22q11.21 in sample EC13HB01069 and sample EC13HB01084 and one case of 22q11.2 distal deletion syndrome attributed to the deletion of 22q11.21‐22q11.23 in sample EC13HB01084; one case of Chromosome 18p deletion syndrome caused by the deletion of 18p11.32‐18p11.31 in sample EC13BD00154; one case of 15q11‐q13 duplication syndrome resulted from the duplication of 15q11.2‐15q13.1 in sample EC16BE00322; one case of 1q21.1 microdeletion syndrome resulted from the deletion of 1q21.1‐1q21.2 in sample EC13HB01354; one case of Prader‐Willi/Angelman syndrome due to the deletion in 15q11.2‐15q11.2 (EC178L04039) and finally the 58.5‐Mb deletion in Xp22.33‐Xp11.1 (EC13CD00028) which could give rise to multiple diseases including Leri‐Weill dyschondrostosis (LWD) ‐ SHOX deletion, Steroid sulphatase deficiency (STS) (Table 2).

Within the 36 CNV cases identified by NIPSCCD, the fetal fraction of male pregnancies varied between 3.77% and 16.02% (Tables 2 and 3). In comparison with the amniocytes testing results, the chromosomal locations of 29 CNVs identified by NIPSCCD could completely cover or overlap at least 51% region of the detected variants in the corresponding amniocytes testing results. All the 12 pathogenic CNVs were detected by both methods, which accounted for 3.3% (12/36) of the total CNVs identified by NIPSCCD. However, among 13 samples containing only one CNV in amniocytes testing data, NIPSCCD had missed a 1.75‐Mb duplication in EC13AK00635 (Table 3) and gained an extra 0.75‐Mb duplication in sample EC13JD00020 (Table 3). In nine samples with multiple CNVs, three of which (EC13BK00003, EC13SA00154, EB13EX00305) produced identical results, whereas the rest of six samples had inconsistence in one or two CNVs (Table 2). Collectively, 7 CNVs detected by NIPSCCD were absent in the amniocentesis test.

Based on the above results and the follow‐up data, the performance of the NIPSCCD method in CNVs identification were evaluated under the following conditions: (a) CNVs identified by both NIPSCCD and amniocytes tests were treated as true positive results; (b) CNVs detected by amniocytes testing but not by NIPSCCD were considered as false negative results; (c) CNVs detected by NIPSCCD but not by amniocytes testing were classified as false positive results. Our analysis showed that for CNVs larger than 10 Mb, the sensitivity, positive prediction value (PPV), and false negative rate (FNR) of NIPSCCD were 91.67%, 84.62%, and 9.33%, respectively, and for CNVs ranged between 5 Mb to 10 Mb, it was 100.00%, 71.43%, and 0, whereas for CNVs less than 5 Mb in size, the sensitivity was 68.42% with the PPV of 81.25% and FNR of 31.58%. Collectively, the NIPSCCD generated an overall sensitivity of 80.56%, PPV of 80.56% and FNR of 19.44% in fetal CNVs detection (Table 4).

**Table 4:** Evaluation of the NIPSCCD method in detecting CNVs

CNV size	TP	FP	FN	Sensitivity (%)	PPV (%)	FNR (%)
>10 Mb	11	2	1	91.67	84.62	9.33
5 Mb–10 Mb	5	2	0	100.00	71.43	NA
<5 Mb	13	3	6	68.42	81.25	31.58
Total CNVs	29	7	7	80.56	80.56	19.44

Note

TP, true positive NIPSCCD‐detected CNVs that were confirmed by amniocytes testing; FP, inconsistent CNVs that were detected by NIPSCCD while not detected by amniocytes testing were classified as false positive; FN, amniocytes testing‐characterized CNVs that were not detected by the NIPSCCD method; PPV, positive predictive value; FNR, false negative rate; NA, not applicable.

### 3.3 Evaluation of NIPSCCD for fetal chromosomal aneuploidy detection

On top of the CNVs detection, we also evaluated the performance of NIPSCCD in chromosomal aneuploidy screening. Among the 20,003 samples, NIPSCCD test identified 124 (0.62%) positive trisomy cases, including 100 cases with trisomy 21, 19 cases with trisomy 18, and 5 cases with trisomy 13, in which 103 cases were confirmed by karyotyping analysis, whereas 21 cases (14 cases of trisomy 21, 4 of trisomy 18, and 3 of trisomy 13) had inconsistent results and considered as false positives (Table 5). The sensitivity and specificity of the NIPSCCD method in fetal aneuploidy detection were calculated and the results showed that the sensitivity of trisomy 13, 18, and 21 detection was 100.00% and the specificity was all higher than 99.9% (Table 5). These results suggested that the NIPSCCD method had high sensitivity and specificity in detecting fetal chromosomal aneuploidy.

**Table 5:** Evaluation of NIPSCCD method in detecting fetal chromosomal aneuploidies

Trisomy	Karyotyping	NIPSCCD results	TP	FP	FN	TN	Sensitivity (%)	Specificity (%)
T21	86	100	86	14	0	19,903	100.00	99.93
T18	15	19	15	4	0	19,984	100.00	99.98
T13	2	5	2	3	0	19,998	100.00	99.99
Total	103	124	103	21	0	19,879	100.00	99.89

Note

TP, true positive; FP, false positive; FN, false negative; TN, true negative.

## 4 DISCUSSION

The widespread availability of cell‐free DNA sequencing based NIPT for fetal aneuploidies using maternal plasma provided an accurate and effective approach to reduce the risks and maternal anxiety that resulted from conventional invasive diagnosis procedures such as amniocentesis and chorionic villus sampling (CVS). There is no doubt that the increasing scope of NIPT from the screening of chromosomal aneuploidies to CNVs would benefit the pregnant women given that the invasive prenatal testing followed by karyotyping and/or chromosomal microarray analysis have been considered as gold standard for CNV analysis (Gregg et al., 2016; Wapner et al., 2012). Although NIPT has been largely focused on chromosomal aneuploidies, its involvement in detecting subchromosomal CNVs is theoretically feasible provided that the fetal fraction involved in the imbalance regions is adequate. For any given CNVs, the detection efficiency is mainly determined by fetal fraction, CNVs size, sequencing coverage, and biological and technical variability of the CNV regions (Zhao et al., 2015) and increasing sequencing depth could significantly improve the sensitivity of CNVs detection (Lo et al., 2016; Yin et al., 2015). However, taken the patients’ affordability into consideration, the deep sequencing‐based methods for noninvasive testing of CNVs (Benn & Cuckle, 2014; Peters et al., 2011; Srinivasan et al., 2013; Yu et al., 2013) are impractical for large‐scaled clinical implementation. Hence, a compromise should be adopted to enable high performance of CNVs detection with NIPT while retaining the cost‐effectiveness.

**Figure 2:**
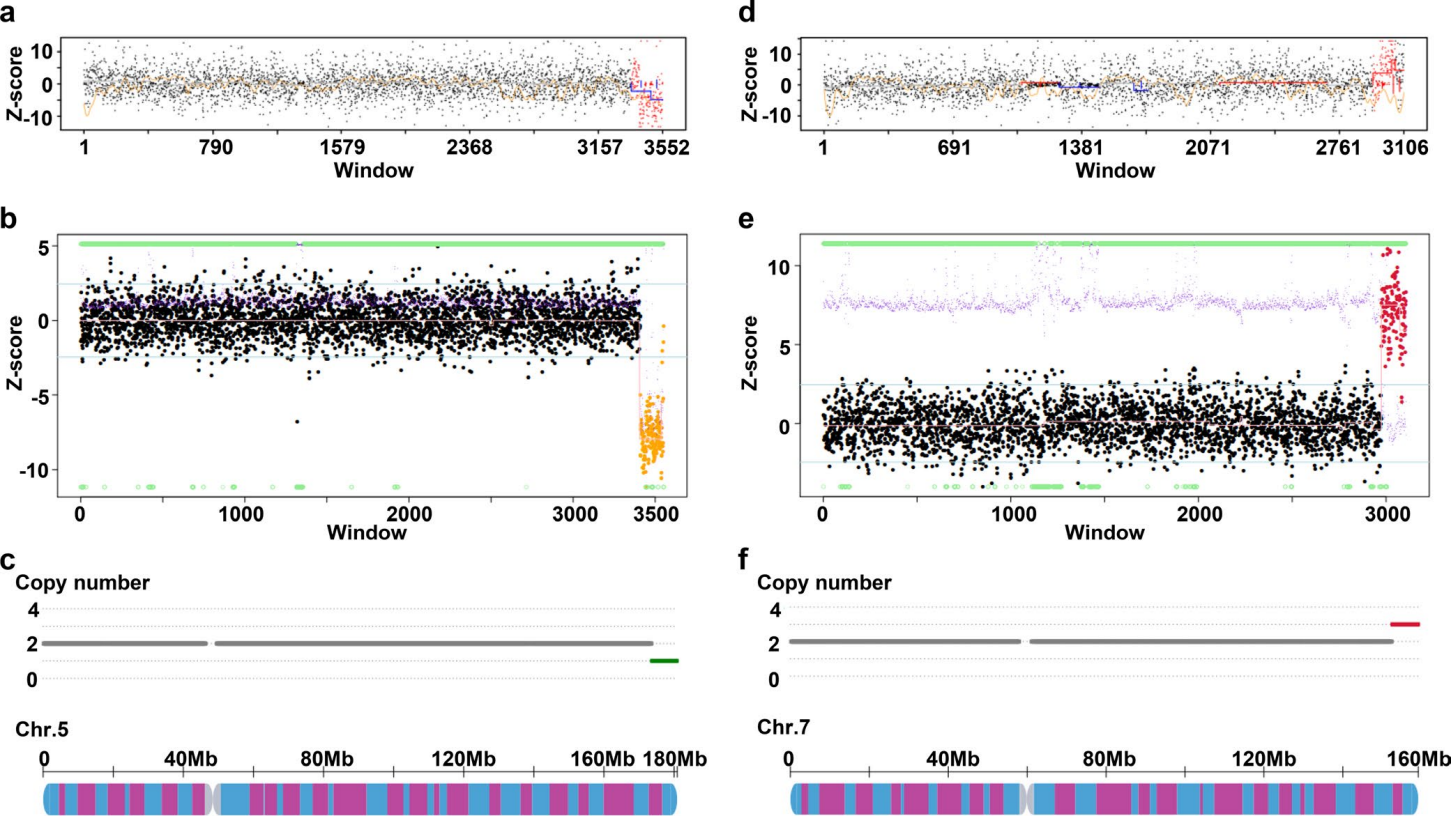
Example of fetal CNVs detection by NIPSCCD. (a) and (d), the NIPSCCD method detected one deletion (a) and one duplication (d) in sample EC13BK00001 respectively. (b) and (e), the amniocytes testing results of sample EC13BK00001 confirmed the deletion and duplication identified by NIPSCCD. (c) and (f), the schematic diagram of the deletion on chromosome 5 (c) and the duplication on chromosome 7 (f) in sample EC13BK00001

In this study, we developed an analytical pipeline, named NIPSCCD (Figure 1), to detect genome‐wide CNVs using low‐pass sequencing of maternal plasma without the prior knowledge of CNV locations and evaluated its performance with 20,003 clinical samples. Till now, most of the studies assessed the performance of NIPT for CNVs with samples containing single CNV, which might be detached from the real clinical environment. We, however, tested the efficacy of NIPSCCD with clinical samples containing but not limited to a single CNV, and the results suggested a robust detection power against samples with multiple CNVs (Table 2; Figure 2). With an average read count of 4.2 M, NIPSCCD could detect CNVs with a minimum size of 0.36 Mb (Table 2) and generated the sensitivity of 91.67% for CNVs > 10 Mb, 100% for CNVs ranged between 10 Mb and 5 Mb, and 68.42% for CNVs <5 Mb (Table 4). The observation of lower sensitivity in CNVs >10 Mb subgroup was attributed to the missed 97.2‐Mb CNV, which resulted in the only false negative case and decreased the sensitivity to 91.67%. Given the large size of this false negative CNV in NIPSCCD test, it is less likely to be the consequence of maternal CNV interference. Since we did not obtain the placenta for further analysis, we proposed that the missed 97.2‐Mb duplication in NIPSCCD, which covered the whole long arm of chromosome X, was resulted from confined placental mosaicism. Comparing with the calling pipeline which generated a sensitivity of 20% in detecting CNVs less than 6 Mb (Lo et al., 2016), NIPSCCD has showed an obvious increase in sensitivity. Also, with a similar read count, NIPSCCD exhibited noticeable improvement than the SSP‐based method proposed by Yin et al. in detecting CNVs less than 5 Mb in size (Yin et al., 2015). Due to the better performance in small CNVs detection, NIPSCCD generated an even higher overall sensitivity for CNVs detection than the SSP‐based method. Moreover, in contrast to the FCAPS method (Liu et al., 2016), NIPSCCD achieved higher performance in detecting large CNVs (>10 Mb) and CNVs between 5 Mb and 10 Mb at a much lower sequencing depth.

Researches over past few years have showed the capability of NIPT for CNVs detection (Benn & Cuckle, 2014; Lo et al., 2016; Peters et al., 2011; Srinivasan et al., 2013; Straver et al., 2014), but most of these methods were not incorporated into the routine aneuploidy test except for WISECONDOR (Straver et al., 2014). However, the WISECONDOR method also had limited clinical value in CNVs detection unless the detection resolution is significantly improved. In this study, we demonstrated that the NIPSCCD method can be incorporated into the detection of fetal aneuploidies. With the testing of 20,003 clinical samples, we achieved an overall 100.00% sensitivity and 99.89% specificity for trisomy 18/13/21 screening (Table 5), suggesting that NIPSCCD has an even slightly higher performance than previous studies (Chiu et al., 2008; Gil, Accurti, Santacruz, Plana, & Nicolaides, 2017; Gil, Quezada, Revello, Akolekar, & Nicolaides, 2015; Hartwig et al., 2018; Zhang, Gao, et al., 2015). Consequently, the NIPSCCD can detect both the chromosomal aneuploidy and subchromosomal CNVs with a single run of low‐pass sequencing without extra sequencing cost.

There were also several limitations in the study. First, in consistent with previous studies, CNV size remained to be a key factor affecting the performance of NIPT subchromosomal abnormalities screening in our work (Chen et al., 2013; Liu et al., 2016; Lo et al., 2016; Yin et al., 2015). The detection power is dramatically decreased with the reduction of CNVs length, and six of the seven false negative CNVs were less than 2.5 Mb (Table 3 & Table 4). Therefore, further optimization of NIPSCCD would be indispensable in the future study to decrease the false negative rate. Second, 36 women with NIPSCCD‐identified CNVs with unknown clinical significance refused to carry out invasive procedure for validation in this study, which could lead to an increase in CNV detection sensitivity as well as the decrease in false positives rate. Similar effect may be observed in the CNV subgroup between 10 Mb and 5 Mb (Table 4). With limited cases of CNVs identified in the subgroup between 10 Mb and 5 Mb, cases bias may have contributed to the higher sensitivity that was observed, and further studies with larger patient population could give a better understanding of the performance of NIPSCCD in CNV detection in clinical setting. Third, most of the positive CNVs were validated by low‐pass whole‐genome sequencing of amniocytes rather than chromosomal microarray analysis. Although many studies have evidenced the capacity of low‐path whole‐genome sequencing in clinical diagnose for subchromosomal CNVs (Dong et al., 2016; Qi et al., 2018; Wang et al., 2018), we could not rule out the potential that confirmatory tests with microarray analysis may impact the false positive and/or negative cases given that the former method is sequencing depth sensitive. Moreover, lack of independent method to calculate fetal fractions for female fetuses in this study, together with the fact that NIPT is incapable of differentiating the maternal CNVs from fetus originated CNVs, the existence of false positive CNVs in our study suggested that NIPSCCD‐detected CNVs should be further confirmed with diagnosis testing or maternal background testing to provide comprehensive information for the posttest genetic counseling.

In summary, our results showed that, under the real clinical environment, the low‐pass WGS‐based NIPSCCD approach had good performance in detecting fetal subchromosomal CNVs >10 Mb and improved efficacy in detecting small CNVs. As the NIPSCCD method can be easily incorporated into the routine screening of trisomy 21/18/13 with high sensitivity and specificity, our study highlighted the prospect of a universal and practical noninvasive prenatal testing of fetal subchromosomal CNVs and chromosomal aneuploidies in a single run of low‐pass sequencing of maternal plasma.

## CONFLICT OF INTEREST

The authors declare that they have no conflict of interest.

## References

[mgg3674-bib-0001] Advani, H. V. , Barrett, A. N. , Evans, M. I. , & Choolani, M. (2017). Challenges in non‐invasive prenatal screening for sub‐chromosomal copy number variations using cell‐free DNA. Prenatal Diagnosis, 37(11), 1067–1075. 10.1002/pd.5161 28950403

[mgg3674-bib-0002] Benn, P. , & Cuckle, H. (2014). Theoretical performance of non‐invasive prenatal testing for chromosome imbalances using counting of cell‐free DNA fragments in maternal plasma. Prenatal Diagnosis, 34(8), 778–783. 10.1002/pd.4366 24676912

[mgg3674-bib-0003] Benn, P. , Cuckle, H. , & Pergament, E. (2013). Non‐invasive prenatal testing for aneuploidy: Current status and future prospects. Ultrasound in Obstetrics and Gynecology, 42(1), 15–33. 10.1002/uog.12513 23765643

[mgg3674-bib-0004] Chen, S. , Lau, T. K. , Zhang, C. , Xu, C. , Xu, Z. , Hu, P. , … Zhang, X. (2013). A method for noninvasive detection of fetal large deletions/duplications by low coverage massively parallel sequencing. Prenatal Diagnosis, 33(6), 584–590. 10.1002/pd.4110 23592436

[mgg3674-bib-0005] Chiu, R. w. k. , Chan, K. c. a. , Gao, Y. , Lau, V. y. m. , Zheng, W. , Leung, T. y. , … Lo, Y. m. d. (2008). Noninvasive prenatal diagnosis of fetal chromosomal aneuploidy by massively parallel genomic sequencing of DNA in maternal plasma. Proceedings of the National Academy of Sciences of the United States of America, 105(51), 20458–20463. 10.1073/pnas.0810641105 19073917 PMC2600580

[mgg3674-bib-0006] Dong, Z. , Zhang, J. , Hu, P. , Chen, H. , Xu, J. , Tian, Q. i. , … Xu, Z. (2016). Low‐pass whole‐genome sequencing in clinical cytogenetics: A validated approach. Genetics in Medicine, 18(9), 940–948. 10.1038/gim.2015.199 26820068

[mgg3674-bib-0007] Fan, H. C. , Blumenfeld, Y. J. , Chitkara, U. , Hudgins, L. , & Quake, S. R. (2008). Noninvasive diagnosis of fetal aneuploidy by shotgun sequencing DNA from maternal blood. Proceedings of the National Academy of Sciences of the United States of America, 105(42), 16266–16271. 10.1073/pnas.0808319105 18838674 PMC2562413

[mgg3674-bib-0008] Gil, M. M. , Accurti, V. , Santacruz, B. , Plana, M. N. , & Nicolaides, K. H. (2017). Analysis of cell‐free DNA in maternal blood in screening for aneuploidies: Updated meta‐analysis. Ultrasound in Obstetrics and Gynecology, 50(3), 302–314. 10.1002/uog.17484 28397325

[mgg3674-bib-0009] Gil, M. M. , Quezada, M. S. , Revello, R. , Akolekar, R. , & Nicolaides, K. H. (2015). Analysis of cell‐free DNA in maternal blood in screening for fetal aneuploidies: Updated meta‐analysis. Ultrasound in Obstetrics and Gynecology, 45(3), 249–266. 10.1002/uog.14791 25639627

[mgg3674-bib-0010] Goldenberg, P. (2018). An update on common chromosome microdeletion and microduplication syndromes. Pediatric Annals, 47(5), e198–e203. 10.3928/19382359-20180419-01 29750287

[mgg3674-bib-0011] Gregg, A. R. , Skotko, B. G. , Benkendorf, J. L. , Monaghan, K. G. , Bajaj, K. , Best, R. G. , … Watson, M. S. (2016). Noninvasive prenatal screening for fetal aneuploidy, 2016 update: A position statement of the American College of Medical Genetics and Genomics. Genetics in Medicine, 18(10), 1056–1065. 10.1038/gim.2016.97 27467454

[mgg3674-bib-0012] Hartwig, T. S. , Ambye, L. , Werge, L. , Weiergang, M. K. , Norgaard, P. , Sorensen, S. , & Jorgensen, F. S. (2018). Non‐invasive prenatal testing (NIPT) in pregnancies with trisomy 21, 18 and 13 performed in a public setting – Factors of importance for correct interpretation of results. European Journal of Obstetrics, Gynecology, and Reproductive Biology, 226, 35–39. 10.1016/j.ejogrb.2018.04.042 29804026

[mgg3674-bib-0013] Li, H. , & Durbin, R. (2009). Fast and accurate short read alignment with Burrows‐Wheeler transform. Bioinformatics, 25(14), 1754–1760. 10.1093/bioinformatics/btp324 19451168 PMC2705234

[mgg3674-bib-0014] Liang, D. , Lv, W. , Wang, H. , Xu, L. , Liu, J. , Li, H. , … Wu, L. (2013). Non‐invasive prenatal testing of fetal whole chromosome aneuploidy by massively parallel sequencing. Prenatal Diagnosis, 33(5), 409–415. 10.1002/pd.4033 23299662

[mgg3674-bib-0015] Liu, H. , Gao, Y. a. , Hu, Z. , Lin, L. , Yin, X. , Wang, J. , … Wang, W. (2016). Performance evaluation of nipt in detection of chromosomal copy number variants using low‐coverage whole‐genome sequencing of plasma DNA. PlOS One, 11(7), e0159233 10.1371/journal.pone.0159233 27415003 PMC4945049

[mgg3674-bib-0016] Lo, K. K. , Karampetsou, E. , Boustred, C. , McKay, F. , Mason, S. , Hill, M. , … Chitty, L. S. (2016). Limited clinical utility of non‐invasive prenatal testing for subchromosomal abnormalities. American Journal of Human Genetics, 98(1), 34–44. 10.1016/j.ajhg.2015.11.016 26708752 PMC4716686

[mgg3674-bib-0017] Lo, Y. M. , Corbetta, N. , Chamberlain, P. F. , Rai, V. , Sargent, I. L. , Redman, C. W. , & Wainscoat, J. S. (1997). Presence of fetal DNA in maternal plasma and serum. Lancet, 350(9076), 485–487. 10.1016/S0140-6736(97)02174-0 9274585

[mgg3674-bib-0018] Minear, M. A. , Lewis, C. , Pradhan, S. , & Chandrasekharan, S. (2015). Global perspectives on clinical adoption of NIPT. Prenatal Diagnosis, 35(10), 959–967. 10.1002/pd.4637 26085345 PMC5065727

[mgg3674-bib-0019] Nevado, J. , Mergener, R. , Palomares‐Bralo, M. , Souza, K. R. , Vallespín, E. , Mena, R. , … Lapunzina, P. (2014). New microdeletion and microduplication syndromes: A comprehensive review. Genetics and Molecular Biology, 37(1 Suppl), 210–219. 10.1590/S1415-47572014000200007 24764755 PMC3983590

[mgg3674-bib-0020] Peters, D. , Chu, T. , Yatsenko, S. A. , Hendrix, N. , Hogge, W. A. , Surti, U. , … Rajkovic, A. (2011). Noninvasive prenatal diagnosis of a fetal microdeletion syndrome. New England Journal of Medicine, 365(19), 1847–1848. 10.1056/NEJMc1106975 22070496 PMC4308687

[mgg3674-bib-0021] Qi, H. , Xuan, Z.‐L. , Du, Y. , Cai, L.‐R. , Zhang, H. , Wen, X.‐H. , … Liang, J.‐B. (2018). High resolution global chromosomal aberrations from spontaneous miscarriages revealed by low coverage whole genome sequencing. European Journal of Obstetrics, Gynecology, and Reproductive Biology, 224, 21–28. 10.1016/j.ejogrb.2018.03.008 29525519

[mgg3674-bib-0022] Rose, N. C. , Benn, P. , & Milunsky, A. (2016). Current controversies in prenatal diagnosis 1: Should NIPT routinely include microdeletions/microduplications? Prenatal Diagnosis, 36(1), 10–14. 10.1002/pd.4710 26492631

[mgg3674-bib-0023] Song, Y. , Liu, C. , Qi, H. , Zhang, Y. , Bian, X. , & Liu, J. (2013). Noninvasive prenatal testing of fetal aneuploidies by massively parallel sequencing in a prospective Chinese population. Prenatal Diagnosis, 33(7), 700–706. 10.1002/pd.4160 23703459

[mgg3674-bib-0024] Srinivasan, A. , Bianchi, D. W. , Huang, H. , Sehnert, A. J. , & Rava, R. P. (2013). Noninvasive detection of fetal subchromosome abnormalities via deep sequencing of maternal plasma. American Journal of Human Genetics, 92(2), 167–176. 10.1016/j.ajhg.2012.12.006 23313373 PMC3567270

[mgg3674-bib-0025] Straver, R. , Sistermans, E. A. , Holstege, H. , Visser, A. , Oudejans, C. B. , & Reinders, M. J. (2014). WISECONDOR: Detection of fetal aberrations from shallow sequencing maternal plasma based on a within‐sample comparison scheme. Nucleic Acids Research, 42(5), e31 10.1093/nar/gkt992 24170809 PMC3950725

[mgg3674-bib-0026] Taylor‐Phillips, S. , Freeman, K. , Geppert, J. , Agbebiyi, A. , Uthman, O. A. , Madan, J. , … Clarke, A. (2016). Accuracy of non‐invasive prenatal testing using cell‐free DNA for detection of Down, Edwards and Patau syndromes: A systematic review and meta‐analysis. BMJ Open, 6(1), e010002 10.1136/bmjopen-2015-010002 PMC4735304 26781507

[mgg3674-bib-0027] Venkatraman, E. S. , & Olshen, A. B. (2007). A faster circular binary segmentation algorithm for the analysis of array CGH data. Bioinformatics, 23(6), 657–663. 10.1093/bioinformatics/btl646 17234643

[mgg3674-bib-0028] Wapner, R. J. , Babiarz, J. E. , Levy, B. , Stosic, M. , Zimmermann, B. , Sigurjonsson, S. , … Benn, P. (2015). Expanding the scope of noninvasive prenatal testing: Detection of fetal microdeletion syndromes. American Journal of Obstetrics and Gynecology, 212(3), 332 e331–339 10.1016/j.ajog.2014.11.041 25479548

[mgg3674-bib-0029] Wapner, R. J. , Martin, C. L. , Levy, B. , Ballif, B. C. , Eng, C. M. , Zachary, J. M. , … Jackson, L. (2012). Chromosomal microarray versus karyotyping for prenatal diagnosis. New England Journal of Medicine, 367(23), 2175–2184. 10.1056/NEJMoa1203382 23215555 PMC3549418

[mgg3674-bib-0030] Weise, A. , Mrasek, K. , Klein, E. , Mulatinho, M. , Llerena, J. C. , Hardekopf, D. , … Liehr, T. (2012). Microdeletion and microduplication syndromes. Journal of Histochemistry and Cytochemistry, 60(5), 346–358. 10.1369/0022155412440001 22396478 PMC3351230

[mgg3674-bib-0031] Yin, A.‐H. , Peng, C.‐F. , Zhao, X. , Caughey, B. A. , Yang, J.‐X. , Liu, J. , … Zhang, K. (2015). Noninvasive detection of fetal subchromosomal abnormalities by semiconductor sequencing of maternal plasma DNA. Proceedings of the National Academy of Sciences of the United States of America, 112(47), 14670–14675. 10.1073/pnas.1518151112 26554006 PMC4664371

[mgg3674-bib-0032] Yin, X. , Du, Y. , Zhang, H. , Wang, Z. , Wang, J. , Fu, X. , … Zhang, X. (2018). Identification of a de novo fetal variant in osteogenesis imperfecta by targeted sequencing‐based noninvasive prenatal testing. Journal of Human Genetics, 63(11), 1129–1137. 10.1038/s10038-018-0489-9 30131598

[mgg3674-bib-0033] Yu, S. C. Y. , Jiang, P. , Choy, K. W. , Chan, K. C. A. , Won, H.‐S. , Leung, W. C. , … Chiu, R. W. K. (2013). Noninvasive prenatal molecular karyotyping from maternal plasma. PlOS One, 8(4), e60968 10.1371/journal.pone.0060968 23613765 PMC3629174

[mgg3674-bib-0034] Zarrei, M. , MacDonald, J. R. , Merico, D. , & Scherer, S. W. (2015). A copy number variation map of the human genome. Nature Reviews: Genetics, 16(3), 172–183. 10.1038/nrg3871 25645873

[mgg3674-bib-0035] Zhang, H. , Gao, Y. , Jiang, F. , Fu, M. , Yuan, Y. , Guo, Y. , … Wang, W. (2015). Non‐invasive prenatal testing for trisomies 21, 18 and 13: Clinical experience from 146,958 pregnancies. Ultrasound in Obstetrics and Gynecology, 45(5), 530–538. 10.1002/uog.14792 25598039

[mgg3674-bib-0036] Zhang, H. , Zhao, Y.‐Y. , Song, J. , Zhu, Q.‐Y. , Yang, H. , Zheng, M.‐L. , … Qiao, J. (2015). Statistical approach to decreasing the error rate of noninvasive prenatal aneuploid detection caused by maternal copy number variation. Scientific Reports, 5, 16106 10.1038/srep16106 26534864 PMC4632076

[mgg3674-bib-0037] Zhao, C. , Tynan, J. , Ehrich, M. , Hannum, G. , McCullough, R. , Saldivar, J.‐S. , … Deciu, C. (2015). Detection of fetal subchromosomal abnormalities by sequencing circulating cell‐free DNA from maternal plasma. Clinical Chemistry, 61(4), 608–616. 10.1373/clinchem.2014.233312 25710461

